# Functional metagenomics reveals novel β-galactosidases not predictable from gene sequences

**DOI:** 10.1371/journal.pone.0172545

**Published:** 2017-03-08

**Authors:** Jiujun Cheng, Tatyana Romantsov, Katja Engel, Andrew C. Doxey, David R. Rose, Josh D. Neufeld, Trevor C. Charles

**Affiliations:** Department of Biology, University of Waterloo, Waterloo, ON, Canada; Dong-A University, REPUBLIC OF KOREA

## Abstract

The techniques of metagenomics have allowed researchers to access the genomic potential of uncultivated microbes, but there remain significant barriers to determination of gene function based on DNA sequence alone. Functional metagenomics, in which DNA is cloned and expressed in surrogate hosts, can overcome these barriers, and make important contributions to the discovery of novel enzymes. In this study, a soil metagenomic library carried in an IncP cosmid was used for functional complementation for β-galactosidase activity in both *Sinorhizobium meliloti* (*α-Proteobacteria*) and *Escherichia coli* (*γ-Proteobacteria*) backgrounds. One β-galactosidase, encoded by six overlapping clones that were selected in both hosts, was identified as a member of glycoside hydrolase family 2. We could not identify ORFs obviously encoding possible β-galactosidases in 19 other sequenced clones that were only able to complement *S*. *meliloti*. Based on low sequence identity to other known glycoside hydrolases, yet not β-galactosidases, three of these ORFs were examined further. Biochemical analysis confirmed that all three encoded β-galactosidase activity. Lac36W_ORF11 and Lac161_ORF7 had conserved domains, but lacked similarities to known glycoside hydrolases. Lac161_ORF10 had neither conserved domains nor similarity to known glycoside hydrolases. Bioinformatic and structural modeling implied that Lac161_ORF10 protein represented a novel enzyme family with a five-bladed propeller glycoside hydrolase domain. By discovering founding members of three novel β-galactosidase families, we have reinforced the value of functional metagenomics for isolating novel genes that could not have been predicted from DNA sequence analysis alone.

## Introduction

Soils harbour the greatest genetic diversity of any habitats on Earth [1]. Our knowledge of microorganisms comprising soil communities is hampered by cultivation challenges for many microorganisms in these communities [2], although improvements in cultivation methods are addressing this bottleneck [3]. The genomes of metabolically versatile soil microbes are potential sources of biocatalysts for use in various industrial processes. Limited knowledge of links between sequence and function prevent rapid progress in bioinformatics-based systems biology. Metagenomics can be used to explore the collective genetic constituency of environmental microbes, including those that are difficult to culture through conventional microbiological techniques. Sequence-based and function-based strategies are used in metagenomics, depending on the main objectives of the particular study. Sequence-based metagenomics identifies genes by sequence similarity to known database sequences. However, it is difficult, if not impossible, to reliably predict the function of truly novel genes without experimental evidence. Functional screening strategies are based on phenotypic detection of the desired activity, heterologous complementation of host strains, and induced gene expression [2, 4, 5]. These experimental activities have identified novel genes showing little similarity to genes of known function [6–9]. In addition, heterologous complementation screening strategies facilitate simultaneous screening of millions of metagenomic clones. Most functional screens are performed in *Escherichia coli* of the *γ-Proteobacteria*. Because gene expression is often host-dependent [10], multi-host systems increase the likelihood of successful gene expression [5, 11–19].

Glycoside hydrolases (GH) hydrolyze the glycosidic linkages of glycosides and oligosaccharides, and are classified into 131 families based on the similarity of amino acid sequences [20]; http://www.cazy.org/Glycoside-Hydrolases.html). The β-galactosidase (EC 3.2.1.23) enzymes are grouped within GH1, GH2, GH35, GH42, and GH59 families. The hydrolytic activity of β-galactosidase can contribute to various applications, such as reducing the lactose content of dairy products [21], producing bioethanol from cheese whey [22], and as the basis of biosensors for detection of lactose [23]. The associated transgalactosylation activity of β-galactosidases can be used to synthesize galactosylated products [24]. Functional screening of metagenomic libraries resulted in discovery of a GH43 enzyme acting on multiple substrates including lactose [25], cold-adapted or thermostable β-galactosidases [26–28], GH1/GH2 [29–33], two novel β-galactosidases without any similarity to known GHs [7], glycosyltransferase family 4 and β-glycosidase with β-galactosidase activity respectively [34, 35].

In this study, we demonstrate the value of metagenomic cosmid libraries for enzyme discovery. Using lactose as the sole carbon source to support growth of *Sinorhizobium meliloti*, we identified three new β-galactosidases from a cornfield soil library and characterized the biochemical properties of these novel enzymes. In doing this, we revealed novel protein sequence space associations with β-galactosidase activity and substrate specificity.

## Materials and methods

### Bacterial strains, plasmids, cosmids, and growth conditions

Several bacterial strains, plasmids, and cosmids were used in this study (Table 1). All *E*. *coli* strains were grown at 37°C in LB medium (1% tryptone, 0.5% yeast extract, 0.5% NaCl, pH 7.0). All *S*. *meliloti* strains were grown at 30°C in LB supplemented with 2.5 mM CaCl_2_ and 2.5 mM MgSO_4_ (LBmc) [36]. Antibiotics were used at the following final concentrations: streptomycin (Sm, 100 μg/ml for *E*. *coli*, 200 μg/ml for *S*. *meliloti*), neomycin (Nm, 200 μg/ml), rifampicin (Rif, 100 μg/ml), kanamycin (Km, 50 μg/ml), tetracycline (Tc, 20 μg/ml for *E*. *coli*, 10 μg/ml for *S*. *meliloti*), gentamicin (Gm, 10 μg/ml for *E*. *coli*), chloramphenicol (Cm, 10 μg/ml for *E*. *coli*).

**Table 1:** Bacterial strains, plasmids, and cosmids.

Bacteria, plasmids, cosmids	Characteristics	References
***S*. *meliloti***		
Rm1021	SU47 *str-21*, Sm^R^	[37]
RmF728	Rm1021 derivative (*lacEFGZ1K1*), Sm^R^ Nm^R^	[38]
***E*. *coli***		
DH5α	F^*-*^ ϕ80*lacZ*ΔM15 Δ(*lacZYA-argF*) *U169 recA1 endA1 hsdR17 phoA supE44 thi-1 gyrA96 relA1*	[39]
DH5α (Rif^R^)	A spontaneous Rif^R^ mutant of DH5α, Rif^R^	[18]
HB101	F^-^ *supE44 lacY1 ara-14 galK2 xyl-5 mtl-1 leuB6 recA13 rpsL20 thi-1 proA2 hsdSB20*, Sm^R^	[40]
BL21(DE3)pLysS	F^-^ *ompT lon hsdS*_*B*_ *gal dcm* λ(DE3) pLysS, Cm^R^	Novagen
MT616	*pro82 thi-1 endA hsdR17 supE44 recA56* (pRK600), Cm^R^	[36]
**Plasmids and cosmids**		
pET-30a(+)	Expression vector, Km^R^	Novagen
pET-30b(+)	Expression vector, Km^R^	Novagen
pK19mobsacB	Cloning vector, Km^R^	[41]
pSRKGm	pBBR1MCS-5 derivative, Gm^R^	[42]
pRK600	Conjugation helper plasmid, Cm^R^	[36]
pJC8	Low-copy broad-host-range Gateway^®^ entry cosmid, Tc^R^ Gm^R^	[18]
pJC97	pJC98 carrying Lac36W-ORF11, Gm^R^	This work
pJC98	pSRKGm derivative carrying a His-tag region from pET-30b(+), Gm^R^	This work
pJC102	pJC98 carrying Lac161-ORF7, Gm^R^	This work
pTR5	pET-30a(+) carrying Lac161-ORF10, Km^R^	This work

### Functional screening of β-galactosidases

**Fig 1:**
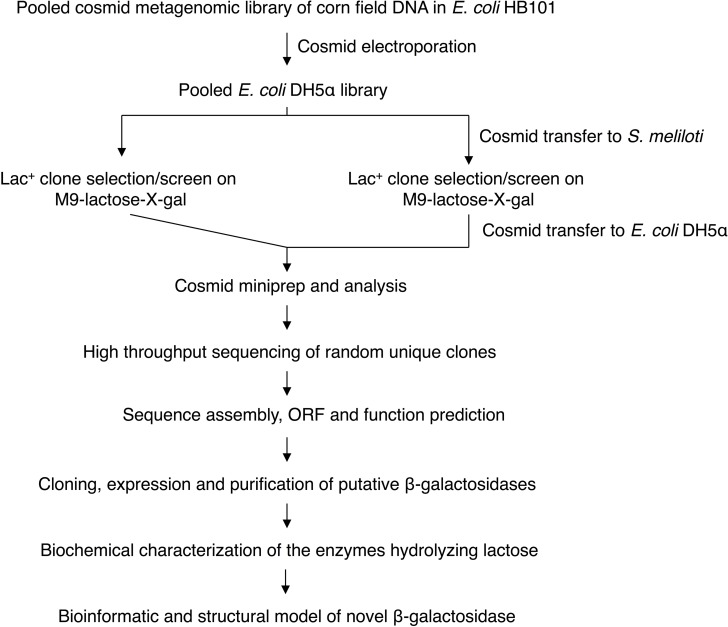
Functional metagenomics selection/screen and characterization of novel β-galactosidases.

The procedure for functional selection and screen of β-galactosidases is illustrated in Fig 1. A previously pooled metagenomic library of corn field soil DNA (12AC; [18]) was used for screening β-galactosidases. The 12AC library contains ~7.9 × 10^4^ cosmid clones with average sizes of 33.4 kb. The cosmid DNA was isolated from the pooled library clones using GeneJET Plasmid Miniprep Kit (Thermo Scientific). *E*. *coli* DH5α (*lacZYA*) was grown in LB to an OD_600_ of 0.6. Cells were collected by centrifugation at 4°C and at 12,300 × *g* for 20 min and then washed three times with cold 10% glycerol. Cells were gently suspended in 2 ml of ice-cold 10% glycerol to about 3 × 10^10^ cells/ml. Electrocompetent cell volumes of 40 μl were mixed with 1 μl of cosmid DNA (45 ng) in a cold 1.5-ml microcentrifuge tube on ice, and then transferred to a cold electroporation cuvette (0.2 cm, Bio-Rad). Electroporation was performed using a Gene Pulser (Bio-Rad; C = 25 μF; PC = 200 ohm; V = 3.0 kV). Liquid SOC medium (1 ml) was added to the cuvette after one pulse. Electroporated cells were transferred to a 1.5-ml microcentrifuge tube and incubated at 37°C in a water bath for 30 min, inverting the tube every 5 min. The tube was then shaken at 200 rpm for 30 min at 37°C. Following concentration by centrifugation, cells were spread on LB Tc plates, and incubated overnight at 37°C. Multiple electroporations were performed to obtain the desired numbers of recombinant *E*. *coli* DH5α clones. Transformants were pooled, then stored at -75°C after addition of DMSO (7% final concentration).

The pooled *E*. *coli* DH5α cosmid clones were washed three times with 0.85% NaCl and then spread on defined M9 medium [43] supplemented with L-arginine (50 μg/ml), thiamine (10 μg/ml), tetracycline (15 μg/ml), lactose (15 mM) as the sole carbon source, and the chromogenic substrate 5-bromo-4-chloro-3-indolyl-β-D-galactopyranoside (X-gal; 36 μg/ml). Plates were incubated at 37°C for 1–3 days. Positive blue colonies were streak purified one time on M9-lactose plates. The Lac^+^ clones were inoculated in 3 ml of LB Tc medium and grown overnight at 37°C. Cosmid DNA was isolated using the GeneJET Plasmid Miniprep Kit (Thermo Scientific), digested simultaneously with EcoRI-BamHI-HindIII, then resolved on 1% agarose gels. Cosmids representative of distinct restriction patterns were retransformed into *E*. *coli* DH5α, and then spread on M9-lactose to confirm the Lac^+^ phenotype.

To screen for Lac^+^ clones in *S*. *meliloti*, 12AC cosmids were conjugated from *E*. *coli* DH5α into *S*. *meliloti* RmF728 (*lacEFGZ1K1*) [38]) with helper plasmid pRK600. The pooled 12AC library clones of 0.25 ml were mixed with 2 ml each of overnight-grown *S*. *meliloti* RmF728 and *E*. *coli* DH5α (pRK600). Cells were collected by centrifugation at 12,300 × *g* for 3 min, washed twice with 2 ml of 0.85% NaCl, and then suspended in 0.5 ml of 0.85% NaCl. Mixed cells were spotted on LB agar and incubated overnight at 30°C. Following collection of the mating spot in a 2-ml microtube and washing twice with 0.85% NaCl, the conjugation mixture was serially diluted and plated on defined M9 medium (Nm Tc) that was supplemented with biotin (0.3 μg/ml), thiamine (10 μg/ml), X-gal (36 μg/ml), and lactose (15 mM) as the sole carbon source. Lac^+^ colonies were streak purified once on M9 lactose plates. Cosmids were then transferred from *S*. *meliloti* to *E*. *coli* DH5α (Rif^R^) via conjugation with the helper plasmid pRK600. *E*. *coli* DH5α carrying the empty cosmid pJC8 was used as a negative control. Lac^+^ cosmid DNA was prepared and analyzed by EcoRI-BamHI-HindIII digestion as described previously.

The Lac^*+*^ phenotypes of *S*. *meliloti* strains were also verified by assaying β-galactosidase activity. Thirty-nine random Lac^+^ strains were grown overnight in LBmc, washed twice with 0.85% NaCl, and then subcultured (1:200 dilution) in M9 lactose medium. Following growth for 48 h, β-galactosidase activity was measured using o-nitrophenyl β-galactoside (ONPG) as described previously [44]. Briefly, whole cells were permeabilized with SDS and incubated with ONPG for 1 h at room temperature. Following termination of the reaction with Na_2_CO_3_, absorption at 420 nm was recorded. Specific activity was represented as (1000 × A_420_)/(time × OD_600_ × culture volume).

### Cloning, expression and purification

The KOD Xtreme DNA polymerase (Novagen) was used for all PCR amplifications with several different primers (S1 Table). PCR amplifications consisted of one cycle of 94°C for 5 min, 30 cycles of 94°C for 30 s, 50–57°C for 30 s, 68°C for 30 s to 3 min, and incubation at 68°C for 10 min. The Lac161_ORF10 was PCR amplified using primers lac161NdeI and lac161HindIII, and cloned into the NdeI-HindIII sites in pET-30a(+) to obtain pTR5. To clone putative GH genes with a C-terminal His tag in a broad-host-range plasmid, a 0.37-kb DNA fragment containing the NdeI site to the end of T7 terminator from pET-30b(+) was amplified using primers JC226 and JC227, and inserted into the NdeI-NheI sites in pSRKGm [42] to obtain plasmid pJC98. The Lac161_ORF7 was obtained by PCR amplification using primers JC220 and JC221, and then inserted into the NdeI-SalI sites in pJC98, yielding pJC102. Lac36W_ORF11 was PCR amplified using primer pair JC212 and JC213, and then cloned into the NdeI-XhoI sites in pJC98 to obtain plasmid pJC97. Plasmids were verified by restriction enzyme digestion analysis.

The expression plasmids pTR5, pJC97, and pJC102 were introduced into *E*. *coli* BL21(DE3)pLysS using the CaCl_2_ transformation method. Gene overexpression was induced by adding 0.1 mM isopropyl β-D-1-thiogalactopyranoside (IPTG) at 20°C for 16 h. Cell pellets were suspended in a lysis buffer (100 mM potassium phosphate; pH 7.4, 5 mM MgSO_4_, 30 μg/ml DNase, 1 mg/ml lysozyme, 2 mM β-mercaptoethanol, and 0.5 mM phenylmethylsulfonyl fluoride), incubated on ice for 30 min, and then disrupted by three passes through EmulsiFlex-C3 (Avestin Inc. Ottawa, Ontario) pressure cell at an internal cell pressure of 1.6 × 10^8^ Pa. His-tagged proteins were purified from supernatants of cell extracts under native conditions using Co^2+^-NTA affinity chromatography (Clontech Laboratories). Purified proteins were dialyzed twice at 4°C against 50 mM potassium phosphate and 10 mM Tris-HCl (pH 7.4).

### Characterization of β-galactosidases

Enzyme activities were measured using a Glucose Oxidase Activity Assay Kit (Sigma-Aldrich) for quantifying the amount of glucose produced upon adding different concentrations of enzyme and lactose. Assays in triplicate were carried out in 96-well microtiter plates The following buffers were used for profiling optimal pH: 100 mM 2-(N-morpholino)ethanesulfonic acid (Mes, pH 5.5–6.5, 100 mM 4-(2-hydroxyethyl)-1-piperazineethanesulfonic acid (pH 7.0–7.5), and 100 mM Tris-HCL (pH 8.0–9.0). Temperature ranged from 20-55ºC. Kinetic parameters were determined with lactose (0.5–15 mM), 100 mM MES buffer of pH 6.5 (Lac161_ORF10) or pH 6.0 (Lac161_ORF7 and Lac36W_ORF11) and enzyme (0–5 mM). Reactions were incubated at 37°C (Lac161_ORF10), 42°C (Lac36W_ ORF11), and 50°C (Lac161_ORF3) for 30 min and terminated with Tris-HCl (pH 7) to a final concentration of 1 M. Aliquots of glucose oxidase/peroxidase reagent (125 μl) were added to each well and left to develop at 37°C for 30 min. Absorbance was measured at 450 nm and compared with a standard glucose curve to determine the amount of glucose released. All reactions were performed in triplicate.

### Bioinformatic analysis

The cosmids were individually indexed and sequenced on Illumina HiSeq 2000 platform. Raw sequence data were assembled as described previously [45]. Open reading frames were annotated using MetaGeneMark [46]. Functions of proteins were predicted by BLAST analysis against NCBI non-redundant protein sequences, Pfam [47], and CAZy analysis toolkit [48]. Transmembrane helices were predicted by the TMHMM Server v. 2.0 (http://www.cbs.dtu.dk/services/TMHMM). Signal peptides were predicted using SignalP 4.0 [49]. Conserved protein domains were searched against NCBI Conserved Domain Database and analyzed with CDTree [50]. Protein structure was predicted with Phyre 2.0 [51]. Taxonomic affiliations of cosmid inserts were assigned based on compositional classifier PhyloPythiaS [52]. Complete sequences of metagenomic Lac^+^ cosmids have been deposited in GenBank with accession numbers KF255992-KF255994 and KF796593-KF796611.

### Protein homology search against metagenomic datasets

SSEARCH36 [53] was used to search 158 metagenomes (32 aquatic, 76 human gut, 50 soil) for homologs to Lac161_ORF7, Lac161_ORF10, and Lac36W_ORF11, with an *E*-value threshold of 0.01. The database of metagenomes was compiled based on the set of aquatic and human gut metagenomes [54, 55], as well as a variety of soil metagenomes obtained from the MG-RAST server (http://metagenomics.anl.gov/). Accession numbers for all datasets are available in S4 Table. For comparison, and to estimate a background level of protein abundance using a housekeeping gene, all metagenomes were also searched for metagenomic homologs of the *rpoB* protein using HMMer (http://hmmer.org/) as implemented in MetAnnotate [56].

## Results

### Functional screening of β-galactosidases

Cosmid clones expressing β-galactosidase genes were screened in previously pooled metagenomic library 12AC [18]. Functional β-galactosidase enzymes can hydrolyse lactose (galactose-β-1,4-glucose) to galactose and glucose, facilitating the growth of bacterial hosts (*lac*) on M9 minimal media when lactose is used as the sole carbon source [18]. Because both the library host *E*. *coli* HB101 (*lacY1*) and surrogate *S*. *meliloti* RmF728 (*lac*) are resistant to streptomycin, which would affect selection of transconjugants in *S*. *meliloti*, 12AC cosmids were transferred from *E*. *coli* HB101 to DH5α (*lacZYA*) via electroporation. We obtained ~8.2 × 10^5^ recombinant clones (Tc^R^) of *E*. *coli* DH5α, which was ~10 fold greater than the number of original cosmid clones in the library. A total of 161 blue colonies were recovered on the selection/screen medium following spreading the *E*. *coli* DH5α clones on M9-lactose plate with X-gal. EcoR1-HindIII-BamHI restriction enzyme digest demonstrated that these 161 clones represented 17 different banding patterns.

We used *S*. *meliloti* from the *α-Proteobacteria* as a soil-dwelling surrogate host for screening in an effort to expand the range of recovered β-galactosidase-encoding clones. *S*. *meliloti* strain RmF728 is a derivative of the well studied Rm1021, and has been modified to carry a genomic deletion that removes the lactose metabolism genes [38]. The 12AC cosmids were transferred from *E*. *coli* DH5α to *S*. *meliloti* RmF728 via *en masse* triparental conjugation, and 1052 Lac^*+*^ colonies that were recovered on M9-lactose medium demonstrated reliable growth after streak purification. The colony color of these clones on M9-lactose containing X-gal ranged from white to varying shades of blue, suggesting that β-galactosidases in those white clones could hydrolyze lactose but not X-Gal. The measurement of β-galactosidase activities of 39 randomly selected *S*. *meliloti* clones grown in M9 lactose medium confirmed that the ability to grow on lactose as sole carbon source was due to cosmid clone-encoded β-galactosidase activity (S2 Table).

Each of the Lac^+^ clones was transferred from *S*. *meliloti* by triparental conjugation to *E*. *coli* DH5α (Rif^R^). Electrophoretic comparison of 291 randomly chosen cosmids digested with EcoRI-HindIII-BamHI demonstrated 208 distinct patterns (71.5%), which suggested that the use of *S*. *meliloti* as a surrogate host for this screen yielded a greater diversity of β-galactosidase genes than when *E*. *coli* was used. There was some overlap with the clones isolated by complementation of *E*. *coli* DH5α, with four restriction enzyme digestion patterns common to both screens. In general, the clones demonstrating a Lac^*+*^ phenotype in both *E*. *coli* and *S*. *meliloti* exhibited higher activity (S2 Table).

### Sequencing and annotation of Lac^+^ cosmids

We chose 3 distinct *E*. *coli* and 22 distinct *S*. *meliloti* Lac^+^ cosmids randomly for high-throughput sequencing (Table 2). Complete insert sequences were successfully assembled from 22 of 25 clones and these sequences and annotated ORFs of the other 22 cosmids were deposited in GenBank (Table 2). The Lac100B, Lac112W, and Lac224 clones from *S*. *meliloti* assembled only partially. Cloned metagenomic inserts were predicted to originate from multiple bacterial phyla (*Cytophaga*, *Thermomicrobia*, *Verrucomicrobia*, *α*-, *β*-, *γ*- and *δ*-*Proteobacteria*) and were GC rich overall (53% to 71%, 64% average).

**Table 2:** 12AC metagenomic clones complementing *E*. *coli* DH5α (*lacZYA*) and *S*. *meliloti* RmF728 (*lacEFGZ1K1*) grown in M9-lactose medium.

Lac^+^ clones ID	Metagenomic DNA (bp)	GC content (%)	Numbers of predicted ORFs	Taxonomic origin^a^	GenBank accession number
Lac13	34,117	63.6	34	*Rhodomicrobium* (*α-Proteobacteria*)	KF796593
Lac16	32,464	65.9	21	*Sphingobium* (*α-Proteobacteria*)	KF796594
Lac20	34,092	60.9	35	*Serratia* (*γ-Proteobacteria*)	KF796595
Lac24B	34,753	61.5	31	*Serratia* (*γ-Proteobacteria*)	KF796596
Lac35B	34,179	61.7	31	*Serratia* (*γ-Proteobacteria*)	KF255992
Lac36B	35,369	61.0	31	*Serratia* (*γ-Proteobacteria*)	KF796597
Lac36W	34,259	67.3	33	*Xanthomonas* (*γ-Proteobacteria*)	KF255993
Lac71	35,712	58.8	28	*Serratia* (*γ-Proteobacteria*)	KF796598
Lac82	34,035	65.8	30	*Sphingopyxis* (*α-Proteobacteria*)	KF796599
Lac84	15,763	65.8	17	*Bradyrhizobium* (*α-Proteobacteria*)	KF796600
Lac100B_102	8,025	52.8	8	*Enterobacteriaceae* (*γ-Proteobacteria*)	KU728997
Lac100B_103	18,097	65.3	19	*Candidatus Accumulibacter* (*β-Proteobacteria*)	KU728998
Lac111	30,066	65.9	23	*Hydrocarboniphaga* (*γ-Proteobacteria*)	KF796601
Lac112W_102	24,084	58.5	18	*Serratia* (*γ-Proteobacteria*)	KU728999
Lac112W_103	13,528	60.6	14	*Serratia* (*γ-Proteobacteria*)	KU729000
Lac121	29,178	64.3	27	*Verrucomicrobia* (*Verrucomicrobiae*)	KF796602
Lac127	31,850	69.9	21	Bacteria	KF796603
Lac146	25,797	62.0	15	*Rhodothermaceae (Cytophagia)*	KF796604
Lac153	35,985	67.4	31	*Xanthomonas* (*γ-Proteobacteria*)	KF796605
Lac160	36,235	69.1	12	*Sphaerobacter* (*Thermomicrobia*)	KF796606
Lac161	35,906	59.1	29	*Chthoniobacter* (*Verrucomicrobia*)	KF255994
Lac172	37,868	59.6	36	*Serratia* (*γ-Proteobacteria*)	KF796607
Lac193	35,861	63.3	25	*Candidatus Methylomirallis*	KF796608
Lac224_103	13,505	68.5	7	*Anaeromyxobacter* (*δ-Proteobacteria)*	KU729001
Lac224_102	4,259	70.9	3	*Myxococcaceae* (*δ-Proteobacteria)*	KU729002
LacEc1	36,404	61.4	31	*Serratia* (*γ-Proteobacteria*)	KF796609
LacEc104	39,079	62.6	37	*Serratia* (*γ-Proteobacteria*)	KF796610
LacEc123	34,035	65.8	34	*Serratia* (*γ-Proteobacteria*)	KF796611

^a ^Taxonomic origin was analyzed using PhylopythiaS.

The *E*. *coli* Lac^+^ clones LacEc1, LacEc104, and LacEc123, and *S*. *meliloti* clones Lac24B, Lac36B, and Lac35B were predicted to originate from *Serratia* of the *γ-Proteobacteria* (Table 2). These clones overlapped over a segment of 15,344 bases (Fig A in S1 File). The 5’ region (positions 3–3,458) exhibited 93% identity to a chromosomal region (positions 2,604,251–2,607,707) of *Serratia marcescens* subsp. *marcescens* Db11 chromosome (GenBank accession HG326223), but the 3’ region (positions 6,652–15,344) of cloned DNA matched best to another region (positions 2,6143,370–2,623,056, 93% identity) of strain Db11. Eleven of 13 ORFs predicted in the overlapping region were 89–98% identical to the clustered orthologs (Fig B in S1 File). The second ORF (Lac35B, GenBank AGW45499) encodes a β-galactosidase (EC 3.2.1.23) with conserved domains of GH2 (Fig 1; Fig B in S1 File). The enzyme matched to the predicted β-galactosidase (SMDB11_2462) of *S*. *marcescens* subsp. *marcescens* Db11 with 98% amino acid sequence identity. Additionally, the annotated β-galactosidase also shares 66% amino acid sequence identity to the well characterized β-galactosidase LacZ (GenBank, BAE76126) of *E*. *coli* K12 substr. W3110. The amino acid residues important for catalytic function in *E*. *coli* LacZ [57] are conserved in the annotated β-galactosidase at Glu^415^, His^417^, Glu^460^, Tyr^502^, and Glu^536^ (S2 File).

Expression of the gene encoding the GH2 β-galactosidase from the cosmid clones in both *E*. *coli* and *S*. *meliloti* suggested a functional promoter(s) upstream of the gene. There were two regions homologous to the conserved -35 and -10 sites of RpoD promoters of *E*. *coli* [58] and *S*. *meliloti* [59] (Fig C in S1 File) in the 102-base intergenic region between the 3’ end (position 23) of an ORF (Lac35B_ORF9, GenBank AGW45500) encoding a two-component response regulator CitA, probably involved in Mg-citrate transport, and the β-galactosidase gene (Lac35B_ORF10, GenBank AGW45499) in Lac35B. These two putative promoters could drive expression of the β-galactosidase gene in *E*. *coli* and *S*. *meliloti*. Unlike the *E*. *coli lac* operon, there was no LacI homolog predicted in the cloned metagenomic DNA of those six cosmids. In addition, expression of the gene encoding β-galactosidase was neither dramatically inhibited by 15 mM glucose nor stimulated by addition of 0.4 mM IPTG in M9 medium, as determined on X-gal plates (S3 File).

Because the lactose permease LacY in *E*. *coli* DH5α [60] and ABC-type transporter LacEFGK1 for lactose in *S*. *meliloti* RmF728 are deleted [38], complementation would require a lactose transporter be encoded within the overlapping region (Fig B in S1 File). We detected an ABC-type transporter system consisting of periplasmic solute-binding protein, permease, and ATP-binding protein (ORF19-ORF17; GenBank AGW45491-AGW45493), but the transporter is probably involved in metal ion uptake. However, ORF21 (GenBank AGW45496) is predicted to encode a major facilitator transporter (IPR020846) with 14 transmembrane helices. This protein belongs to the same major facilitator superfamily as *E*. *coli* lactose permease (LacY) and may be functional as a lactose transporter when expressed in *E*. *coli* DH5α and *S*. *meliloti* RmF728.

The Lac^+^ clones Lac20, Lac71, and Lac172 isolated in *S*. *meliloti* shared a region of 14,707 bp (Fig A and B in S4 File) that was 93% identical to a segment (positions 2,578,724–2,593,427) of the *S*. *marcescens* WW4 chromosome (GenBank accession CP003959). The 14 annotated ORFs within this region exhibited 85–100% amino acid sequence identities to the clustered orthologs (Fig B in S4 File). These data suggest that the cloned DNA in Lac20, Lac71, and Lac172 originated from *γ-Proteobacteria*. One of the two major facilitator transporters (Lac20_ORF31, GenBank accession AHN97675; Lac20_ORF33, GenBank accession AHN97677) might be involved in lactose uptake in *S*. *meliloti*. We were unable to identify an ORF encoding a predicted β-galactosidase based on protein sequence homology.

Examination of the annotated ORFs of the remaining 13 Lac^+^ cosmids from *S*. *meliloti* (Table 2) also did not suggest any candidate that resembled known β-galactosidases. Based on a protein sequence comparison to the CAZy database, which showed low level similarity to proteins carrying known GHs, but not β-galactosidases, we chose Lac36W_ORF11 (GenBank accession AGW45517), Lac161_ORF7 (GenBank accession AGW45552), and Lac161_ORF10 (GenBank accession AGW45555) for biochemical characterization of putative β-galactosidase activity. We amplified the selected ORFs with PCR and cloned the amplicons into expression vectors, generating C-terminal His tags for overexpression in *E*. *coli* and subsequent affinity purification. Following processing, the resulting affinity-purified proteins were assayed for β-galactosidase activity.

### Biochemical characterization of Lac36W_ORF11

Cosmid Lac36W exhibited β-galactosidase activity in *S*. *meliloti* (S2 Table), but not in *E*. *coli*. The cosmid contained a metagenomic DNA fragment of 34,259 bp with 67.3% GC (GenBank accession KF255993). The cloned DNA was assigned taxonomically to *Xanthomonas* of the ɣ-*Proteobacteria* (Table 2).

**Fig 2:**
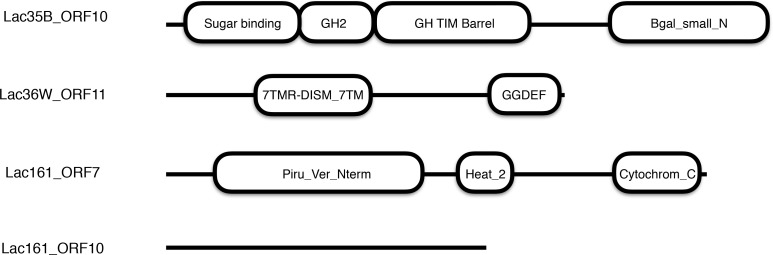
Conserved domains (*E* < 0.01) in β-galactosidases isolated from 12AC metagenomic library clones.

**Fig 3:**
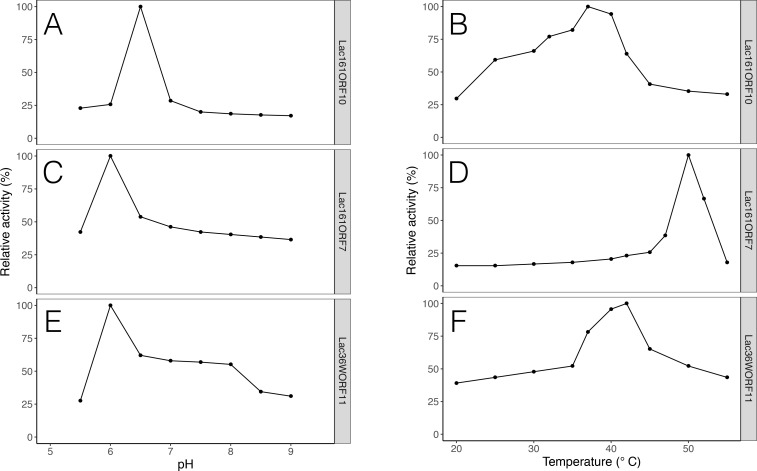
Biochemical characterization of novel β-galactosidases. pH profiles of Lac161_ORF10 (A), Lac161_ORF7 (C), Lac36W_ORF11 (E). Temperature profiles of Lac161_ORF10 (B), Lac161_ORF7 (D), Lac36W_ORF11 (F).

Protein sequence searches of the predicted 33 ORFs against the CAZy database suggested that Lac36W_ORF11 (GenBank accession AGW45517) showed sequence similarity to the protein ERE_21070 of *Eubacterium rectale* M104/1 (GenBank accession CBK94002), which has a Glyco_hydro_53 domain (Genbank accession CBK94002). Lac36W_ORF11 had a signal peptide of 21 amino acids predicted by SignalP 4.0 [49] and two domains (Fig 2): a 7 transmembrane region of receptor with diverse intracellular signaling module (7TMR-DISM-7TM; PF07695), and diguanylate cyclase domain (DGC or GGDEF, PF00990). We predict that the 7TMR-DISM-7TM domain might function as a lactose binding domain like other 7TM-containing proteins [61] and the C-terminal region may act as a β-galactosidase, although it exhibited no similarity to the endo-β-1,4-galactanase domain in the protein ERE_21070 and other known GH family members. Therefore, we cloned the entire Lac36W_ORF11 and expressed it in *E*. *coli*. Purified Lac36W_ORF11 protein was able to hydrolyze lactose to galactose and glucose (Table 3). The enzyme maintained 60% activity in the pH range of 6.5–8.0 (Fig 3E) and still kept ~50% activity at 50°C (Fig 3F). Because there was no similarity to any known GH domain and carbohydrate binding module (CBM), we proposed that Lac36W_ORF11 (GenBank, AGW45517) is a novel β-galactosidase.

**Table 3:** Biochemical characterization of novel β-galactosidases from 12AC Lac^+^ metagenomic clones complementing *S*. *meliloti* RmF728 (*lac*). The β-galactosidase activity of purified proteins was assayed using lactose as substrate. Released glucose was quantified with a glucose oxidase activity kit.

ORFs	Proteins	Molecular weight (kDa)	pI	*K*_*m*_ (mM)	*k*_*cat*_ (s^-1^)	*k*_*cat*_/*K*_*m*_ (s^-1^ M^-1^)	Optimal tempe-rature (°C)	Optimal pH
Lac36W_ORF11	β-Galactosidase (GenBank, AGW45517)	79.6	8.2	2.5	10.4	3.7 × 10^3^	42	6.0
Lac161_ORF7	β-Galactosidase (GenBank, AGW45552)	109.0	6.7	1.8	13.2	7.3 × 10^4^	50	6.0
Lac161_ORF10	β-Galactosidase (GenBank, AGW45555)	63.7	9.1	3.2	8.6	2.7 × 10^3^	37	6.5

The Lac36W_ORF11 was situated within a putative operon, flanked by Lac36W_ORF12, immediately downstream, and Lac36W_ORF10, immediately upstream. Lac36W_ORF12 encodes a putative methionine-S-sulfoxide reductase and is located 107-bp downstream of the Lac36W_ORF11 (Fig A in S5 File). Lac36W_ORF10 was located 5 bp upstream of Lac36W_ORF11 and encodes a hypothetical protein (DUF2007). The nature of the promoter for this predicted operon and its basis for function in *S*. *meliloti*, but not *E*. *coli*, is unclear. The Lac36W_ORF14 is predicted to encode a transcriptional regulator (LysR-like) that may play a role in regulation of the operon. There were no ORFs encoding homologs to known transporters in the cloned 34-kb DNA fragment and, as a result, the genetic basis for uptake of lactose is unknown.

### Biochemical characterization of Lac161_ORF7

Cosmid Lac161 complemented the Lac^-^ phenotype of *S*. *meliloti* RmF728 but could not complement *E*. *coli* DH5α. The cosmid carried an insert of 35,906 bp with 59.1% GC content (GenBank accession KF255994). The metagenomic DNA was assigned taxonomically to *Chthoniobacter* of the phylum *Verrucomicrobia* (Table 2).

Among the 29 annotated ORFs, Lac161_ORF7 (GenBank accession AGW45552) was predicted to be a membrane-bound dehydrogenase protein with three domains (Fig 2): a putative membrane-bound dehydrogenase domain Piru-Ver-Nterm,

TIGR02604), Heat repeat 2 (PF13646), and a cytochrom_C (PF00034) with a putative heme-binding motif CxxCH (TIGER02603). The Heat_2 and Cytochrom_C domains may be involved in intracellular transport and electron transfer. In addition, Lac161_ORF7 was homologous to several proteins that were annotated as probable GHs, such as HVO_B0215 (GenBank accession ADE01485.1; CBM16, CAZy) of *Haloferax volcanii* DS2. Further sequence alignment analysis did not show any similarity to known GHs or CBMs. To determine whether the gene product exhibited any GH activity, the Lac161_ORF7 was cloned and expressed. Purified ORF7 protein was able to hydrolyze lactose with a *K*_*m*_ of 1.8 mM, which is the lowest of the three β-galactosidases studied in this work (Table 3). In addition, the *K*_*m*_ value of Lac161_ORF7 is similar to the reported *K*_*m*_ (2.0) of *E*. *coli* LacZ [62]. The ORF7 protein was most active at the same pH of 6.0 as Lac36W_ORF11 (Table 3; Fig 3C and 3E), but the highest activity of Lac161_ORF7 was observed at 50°C (Fig 3D). In addition, Lac161_ORF7 had the highest *k*_*cat*_/*K*_*m*_ among the β-galactosidases identified in this study. These results implied that Lac161_ORF7 (GenBank accession AGW45552) represents a novel β-galactosidase family.

### Biochemical characterization of Lac161_ORF10

Protein sequence comparison with the Pfam database suggested that Lac161_ORF10 (GenBank accession AGW45555) grouped with a family of proteins of unknown function DUF377 (PF04041; Fig 1), some of which have been predicted to be β-fructosidases (GH32 and GH68), or α*-*L-arabinase and β-xylosidase (GH43 and GH62) [63]. Because of this observation, the Lac161_ORF10 was overexpressed and purified. The resulting gene product was able to hydrolyze lactose with a *K*_*m*_ of 3.2 mM, similar to the values of Lac36W_ORF11 and Lac161_ORF7 (Table 3). The optimal pH and temperature of β*-*galactosidase activity was 6.5 and 37°C, respectively (Fig 3A and 3B). In order to further investigate the range of substrate specificity, four other disaccharides were tested as substrates. When sucrose (glucose-β-1,2-fructose) was added, no glucose was released, suggesting that Lac161_ORF10 was not a β-fructofuranosidase (or invertase, GH32). Additionally, the ORF10 protein was unable to catalyze hydrolysis of xyloside (xylose-β-1,4-xylose, often associated with GH43), maltose (glucose-α-1,4-glucoside, often associated with GH64), and cellobiose (glucose-β-1,4 glucoside, often associated with GH1). Sequence analysis and activity assays therefore suggested that Lac161_ORF10 (Genbank accession AGW45555) is also a new β-galactosidase, like Lac36W_ORF11 (GenBank accession AGW45517) and Lac161_ORF7 (GenBank accession AGW45552) proteins.

Lac161_ORF7 and ORF_10 encoding the two novel β-galactosidases might form one operon along with Lac161_ORF8 and Lac161_ORF9 (Fig B in S4 File). The Lac161_ORF8 encodes a hypothetical protein (GenBank accession AGW45553) homologous to an enolase superfamily including o-succinylbenzoate synthase (cd03320). Lac161_ORF9 encodes a hypothetical protein (GenBank accession AGW45554) with a similar domain to methane oxygenase (PF14100). The reason for gene expression in *S*. *meliloti* but not *E*. *coli* is not yet known.

### Bioinformatic and structural modeling of Lac161_ORF10

**Fig 4:**
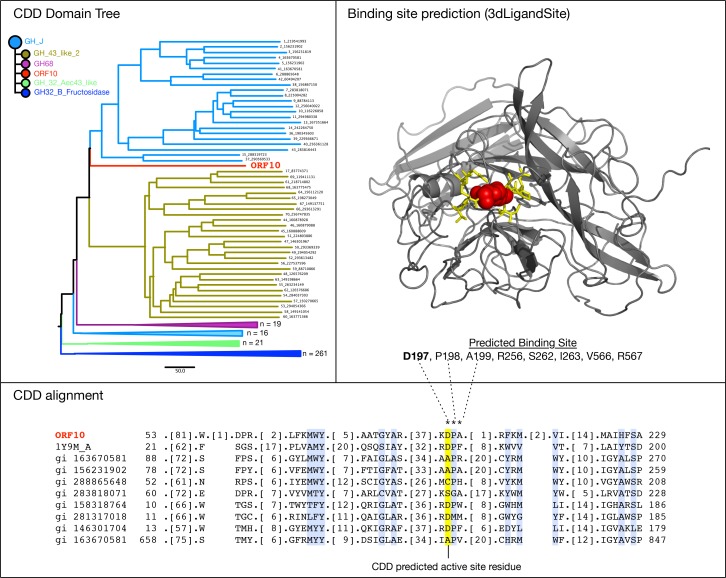
Bioinformatic characterization of a putative glycosyl hydrolase domain in Lac161_ORF10. (A) The NCBI Conserved Domain Database (CDD) predicts Lac161_ORF10 as a divergent member of the GH_J clan of glycosyl hydrolases. (B) Structural model of Lac161_ORF10 generated by Phyre 2.0, with a predicted cluster of 8 ligand-binding residues highlighted in yellow. The putative binding site was predicted by 3dLigandSite based on the Phyre model with PDB ID 1vkd (chain A) as the template. A NAG ligand is shown in red, which approximates the location of a lactose molecule in Lac161_ORF10. (C) An alignment of Lac161_ORF10 with the most similar members of the CDD’s GH_J sequence cluster (Genbank accession numbers are included on the right of the tree). The most conserved columns are coloured light blue. A predicted active site feature (D197) is highlighted in yellow, and is consistent with 3dLigandSite’s predicted cluster of ligand-binding residues.

A search of Lac161_ORF10 (GenBank accession AGW45555) against the NCBI Conserved Domain Database (CDD; [50] revealed no significant hits to characterized protein domains (*E* < 0.01). However, a glycoside hydrolase superfamily domain (GH43_62_32_68 superfamily, cl14647) was detected over region 130–234 as the top-scoring CDD hit overall (*E* = 0.10). More specifically, the match corresponds to a GH_J clan domain, which includes GH32 and GH68 enzymes. The presence of a GH_J domain within Lac161_ORF10 is further supported by the domain architectures of related sequences. The top 10 homologs of Lac161_ORF10 detected by BLAST were mainly from *Bacteroides* (S3 Table), and all possessed this domain over the aligning region (*E* < 0.01). According to CDTree, Lac161_ORF10 represented a highly distinct branch within the GH_J sequence cluster (Fig 4A), which provided some explanation for the observed weak similarity to existing CDD domains.

Proteins within the GH_J superfamily, including GH32 and GH68, all posses a five-bladed propeller fold, and share a funnel-shaped active site typically composed of a catalytic nucleophile (e.g., Asp) and proton donor (e.g., Glu) acting as the general acid/base as well as a RDP motif [64] involved in stabilizing the transition state (Fig 4B). Our analysis suggests that Lac161_ORF10 also shared some of these characteristics.

Using Phyre 2.0 [51], a structural model of Lac161_ORF10 was generated. Phyre predicted a five-bladed propeller fold for Lac161_ORF10 (Fig 4B) with high confidence (99.9%) based on the template PDB 1vkd_A, a predicted glycoside hydrolase from *Thermotoga maritima* (Tmari_1232). Both Tmari_1232 and Lac161_ORF10 are members of the Pfam DUF377 family, further supporting the model. We then analyzed potential active sites using a sequence and structure-based approach. According to the CDD sequence alignment, Lac161_ORF10 possesses a KDP motif (residues 196–198) that aligns to the active site RDP motif in the reference 1y9m structure (Fig 4C). Ligand-binding sites were also predicted in the structural model using 3dLigandSite [65]. This revealed a predicted cluster of eight residues, including the previously identified D-197 residue, as forming the putative active site (Fig 4B). However, alternate alignments and putative active sites from those reported above are possible given the structural repetition of five-bladed propellers. Ultimately, Lac161_ORF10 (GenBank, AGW45555) appears to represent a novel family of β-galactosidase with a GH_J-like five-bladed propeller glycoside hydrolase domain, and an active site similar in composition to other members of this superfamily.

### Metagenome abundance

**Fig 5:**
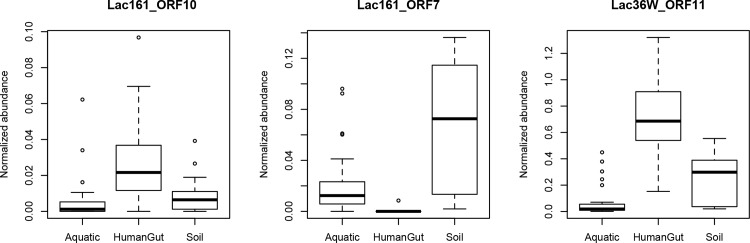
Protein homology searches of novel β*-*galactosidase sequences of Lac161_ORF10, Lac161_ORF7 and Lac36W_ORF11 against aquatic, human gut, and soil metagenomic databases, normalized to the *rpoB* gene.

In order to investigate the distribution of sequences similar to the newly described β-galactosidase sequences throughout different metagenomes, we performed protein homology searches with these sequences against collections of aquatic, human gut, and soil metagenomic databases (Fig 5). The results were normalized against *rpoB* gene abundance. Homologs to each of the three genes were represented in all three habitats. However, Lac36W_ORF11 in the human gut was by far of greatest relative abundance. Lac36W_ORF11 was also high in soil, but not as high as in human gut. Although of overall lower relative abundance, Lac161_ORF10 was also of greater abundance in human gut than in soil or aquatic metagenomes. Lac161_ORF7 exhibited a distinct profile, being extremely rare in the human gut, at low levels in aquatic samples, and at higher levels in soil metagenomes. It will be of interest to determine whether these homologs are also functional β-galactosidases.

## Discussion

Metagenomics provides unprecedented access to the genomic potential of uncultivated microbial communities. Despite enormous progress resulting from developments in high throughput sequencing, the potential for novel enzyme discovery remains highest using a functional metagenomics approach, in which genes are isolated based on their function rather than by DNA sequence similarity to already known genes [9]. Using such an approach, we have discovered genes encoding novel types of lactose hydrolyzing enzymes. The enzymes encoded by these genes were biochemically similar to known enzymes, although they would not have been easily predicted by their sequences without knowing that they were carried on a segment of DNA that encoded β-galactosidase activity. These results demonstrate the importance of sequence-agnostic functional screens for the discovery of enzymes of novel origin, and suggest that further implementation of this strategy will contribute to fundamental knowledge about the relationship between sequence and protein function, improve the resolution of sequence based metagenomics, and expand the repertoire of novel enzymes available for industrial applications.

This work follows on other metagenomic functional screening efforts that have discovered β-galactosidases of GH1 [29, 31], GH2 [31–33], GH3 [66] GH42 [26, 27, 31–33], GH43 [25, 30, 66], GT4 [35], glycosidase [34] and two new GH members [7]. Some of the Lac^+^ clones recovered from *S*. *meliloti* can hydrolyze lactose but not X-Gal, suggestiong that X-Gal was not a good fit with the active sites of those enzymes. The same property of other metagenomic β-galacctosidases has been reported [28]. Here we have highlighted the application of functional metagenomics for mining novel enzymes from soil microbial communities. Although the functional metagenomics strategy has potential for expanding the availability of enzymes that can be further developed for biotech applications, it is perhaps just as important to apply such strategies to the expansion of knowledge that will inform functional interpretation of DNA sequence. This in turn could impact on the ability to derive metabolic information from genome sequence, even from uncultivated microorganisms.

We suggest that the use of a diversity of surrogate hosts for functional metagenomic screening has the potential to substantially extend the breadth of gene discovery. By discovering founding members of three novel β-galactosidase families, we have reinforced the value of functional metagenomics for isolating novel genes that could not have been predicted from DNA sequence analysis alone. Activity-based screening of metagenomic library clones for biocatalysts is dependent on the expression of genes of interest and presence of accessory components required for the enzyme activity in the surrogate hosts [5, 11]. Multi-host-systems have been developed to improve functional screening [12–14, 16, 17, 19, 67]. In the present work, functional screening of the corn field soil library (12AC) for the ability to complement β-galactosidase mutants resulted in a greater number of distinct clones using *S*. *meliloti* than the more widely used *E*. *coli*. In addition, three novel β-galactosidase genes were identified only in *S*. *meliloti*. These data emphasize the importance of developing of multi-host systems for functional screening.

## Supporting information

S1 File Lac^+^ clones isolated from *E*. *coli* (LacEc1, LacEc104 and LacEc123) and *S*. *meliloti* (Lac24B, Lac35B and Lac36B). (A) An overlapping region of 15,344 bp was present in those cosmids. (B) A β-galactosidase of family GH2 (ORF10, solid box), and putative lactose transporter (ORF21, dash lined box) were predicted in Lac35B. The regions encoding orthologs in *γ-Proteobacteria Serratia marcescens* subsp. *marcescens* Db11 chromosome (GenBank HG326223; 2,623,056–2,604,251 nt) were highlighted. (C) Putative RpoD promoters (P) active in both *E*. *coli* and *S*. *meliloti* were located upstream of the β-galactosidase gene. The same enzyme was encoded by LacEc1_ORF31, LacEc104_ORF20, LacEc123_ORF13, Lac24B_ORF9, and Lac36B_ORF3 respectively.
(PDF)

S2 File Sequence alignment of β-glactosidases LacZ of *E*. *coli* K12 substr, W3110 (Genbank, BAE76126) and 12AC metagenomic clone LacEc1 (β-Gal; LacEc1_ORF31; GenBank, KF96609). Conserved amino acids Glu^415^, His^417^, Glu^460^, Tyr^502^ and Glu^536^ at the active sites of LacEc1_ORF31 were highlighted.
(PDF)

S3 File β-Gaclactosidase activity of Lac^+^ clones isolated from *E*. *coli* DH5α. Empty cosmid pJC8 was used as a negative control. X-Gal was used as chromogenic substrate. (A) M9-glucose (15 mM), (B) M9-lactose (15 mM), (C) M9-glycerol (30 mM) + 0.4 mM IPTG.
(PDF)

S4 File Lac^+^ clone Lac20, Lac71 and Lac172 isolated from *S*. *meliloti*. (A) An overlapping region of 14,707 bp was present in those cosmids. (B) The major facilitator transporter(s) (solid box) in the region might be involved in lactose uptake. The hypothetical protein(s) (dash lined box) might be a β-galactosidase. Orthologs in *γ-Proteobacteria Serratia marcescens* WW4 chromosome (GenBank CP003959; 2,578,724–2,593,247 nt) were highlighted.
(PDF)

S5 File A DNA fragment carrying genes encoding β-galactosidases in Lac^+^ cosmids Lac36W and Lac161. (A) A gene locus from cosmid Lac36W (GenBank, KF255993). Lac36W_07, cytosine/adenosine deaminase; Lac36W_08, hypothetical protein; Lac36W_09, glutaminyl-tRNA synthetase; Lac36W_10, hypothetical protein; **Lac36W_11, β-galactosidase**; Lac36W_12, methionine-S-sulfoxide reductase; Lac36W_13, hypothetical protein; Lac36W_14, LysR family transcriptional regulator. The locations of potential promoter regions (P) were showed. (B) A gene locus from cosmid Lac161 (GenBank, KF255994). Lac161_06, histidine kinase; **Lac161_07, β-galactosidase**; Lac161_08, hypothetical protein; Lac161_09, hypothetical protein; **Lac161_10, β-galactosidase**; Lac161_11, hypothetical protein; Lac161_12, host specificity protein. The positions of potential promoter regions (P) are shown.
(PDF)

S1 Table DNA oligonucleotides used in this study with restriction recognition sites underlined. (PDF)

S2 Table β-Galactosidase activities of random 12AC *lac*^+^ clones in *S*. *meliloti* RmF728. (PDF)

S3 Table Top twenty homologs of Lac161_ORF10 detected by a BlastP search of the NCBI nr database. (PDF)

S4 Table Detected abundance of three novel beta-galactosidases in a variety of metagenomic datasets. (PDF)
