# Supplementary material for: Functional metagenomics reveals novel β-galactosidases not predictable from gene sequences
Source: PLoS One. 2017 Mar 8;12(3):e0172545. doi: 10.1371/journal.pone.0172545 (PMC5342196; doi:10.1371/journal.pone.0172545)
Supplement: S1 File — (A) An overlapping region of 15,344 bp was present in those cosmids. (B) A β-galactosidase of family GH2 (ORF10, solid box), and putative lactose transporter (ORF21, dash lined box) were predicted in Lac35B. The regions encoding orthologs in γ-Proteobacteria Serratia marcescens subsp. marcescens Db11 chromosome (GenBank HG326223; 2,623,056–2,604,251 nt) were highlighted. (C) Putative RpoD promoters (P) active in both E. coli and S. meliloti were located upstream of the β-galactosidase gene. The same enzyme was encoded by LacEc1_ORF31, LacEc104_ORF20, LacEc123_ORF13, Lac24B_ORF9, and Lac36B_ORF3 respectively. (PDF) [file pone.0172545.s001.pdf]

A.

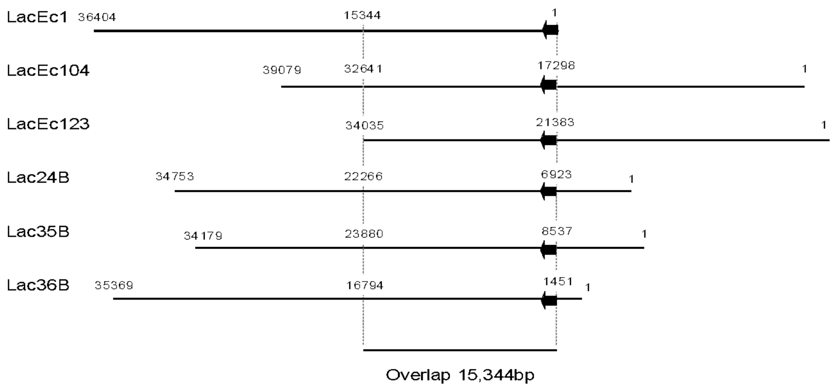

B.

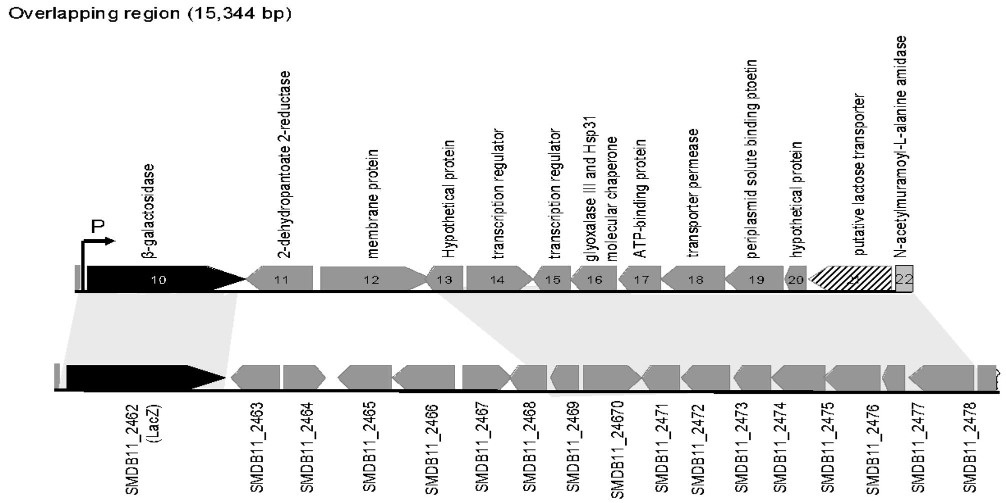

Serratia marcescens sub sp. *marcescens* Db11 chromosome (GenBank HG326223; 2,623,056-2,604,251 nt)

C.

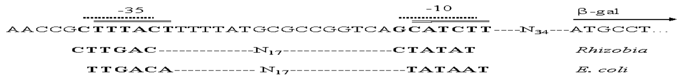

S1 Figure
