# Supplementary material for: Functional metagenomics reveals novel β-galactosidases not predictable from gene sequences
Source: PLoS One. 2017 Mar 8;12(3):e0172545. doi: 10.1371/journal.pone.0172545 (PMC5342196; doi:10.1371/journal.pone.0172545)
Supplement: S2 File — Conserved amino acids Glu415, His417, Glu460, Tyr502 and Glu536 at the active sites of LacEc1_ORF31 were highlighted. (PDF) [file pone.0172545.s002.pdf]

LacZ -MTMITDSLAVVLQRRDWENPGVTQLNRLAAHPPFASWRNSEEARTDRPSQQLRSLNGEW
β-Gal MPAAPLASLTEILARRDWQNPACTHYRRLEAHPPFASWRTVEDARDDAPSASRRSLNGEW

LacZ RFAWFPAPEAVPESWLECDLPEADTVVVPSNWQMHGYDAPIYTNVTYPITVNPPFVPTEN
β-Gal RFNYFPRPEAAPESWLQQDLPDAAPLAVPGNWQLAGYDAPIYTNVRYPFPVDPPRVPEDN

LacZ PTGCYSLTFNVDESWLQEGQTRIIFDGVNSAFHLWCNGRWVGYGQDSRLPSEFDLSAFLR
β-Gal PTGCYSRAFSVDPAWLAAGQTRVIFDGVNSAFYLWCNGHWVGYSQDSRLPAEFDLSPWLR

LacZ AGENRLAVMVLRWSDGSYLEDQDMWRMSGIFRDVSLLHKPTTQISDFHVATRFNDDFSRA
β-Gal PGENRLAVMVLRWCDGSYLEDQDMWRMSGIFRDVSLLHKPAAHLSDVRITTPLHDSFTRG

LacZ --VLEAEVQMCGELRDYLRVTVSLWQGETQVASGTAPFGGEIIDERGGYADRVTLRLNVE
β-Gal ELVVTARANRPGP----LQVQVQLWRDGARVAERIQPLGSEIVDERGAYDDRVTLRLPVE

LacZ NPKLWSAEIPNLYRAVVELHTADGTLIEAEACDVGFREVRIENGLLLLNGKPLLIRGVNR
β-Gal RPALWSAETPTLYRATVALLSPEGEIIEVEAYDVGFRQVEISGGLLKLNGQPLLIRGVNR

415
LacZ HEHHPLHGQVMDEQTMVQDILLMKQNNFNAVRCSHYPNHPLWYTLCDRYGLYVVDEANI**E**
B-Gal HEHHPRHGQVMDEATMRHDILLMKQHNFNAVRCSHYPNHPLWYRLCDRYGLYVVDEANI**E**

417 460
LacZ T**H**GMVPMNRLTDDPRWLPAMSERVTRMVQRDRNHPSVIIWSLGN**E**SGHGANHDALYRWIK
β-Gal T**H**GMQPMNRLADDPLWLPAMSERVTRMVQRDRNHPCIIIWSLGN**E**SGHGANHDALYRWVK

502
LacZ SVDPSRPVQYEGGGADTTATDIICPM**Y**ARVDEDQPFPAVPKWSIKKWLSLPGETRPLILC
β-Gal SQDPTRPVQYEGGGADTAATDIICPM**Y**ARVDQDQPFPAVPKWAIGKWIGLPEEPRPLILC

536
LacZ **E**YAHAMGNSLGGFAKYWQAFRQYPRLQGGFVWDWVDQSLIKYDENGNPWSAYGGDFGDTP
β-Gal **E**YAHAMGNSFGGFERYWRAFHAHPRLQGGFVWDWVDQALIKRDDRGEEFWAYGGDFGDTP

LacZ NDRQFCMNGLVFADRTPHPALTEAKHQQQFFQFRLSGQ--TIEVTSEYLFRHSDNELLHW
β-Gal NDRQFCLNGLVFADRTPHPALFEAQRAQQLFRFAFDAASLTLTVTSDYLFRHTDNEQLNW

LacZ MVALDGKPLASGEVPLDVAPQGK-QLIELPELPQPESAGQLWLTVRVVQPNATAWSEAGH
β-Gal RLELDGVERASGSLDLALPPQGSTRFTLLDRLPMLHQPGELWLNVEVVQPQATDWSEAHH

LacZ ISAWQQWRLAENLSVTLPAASHAIPHLTTSEMDFCIELGNKRWQFNRQSGFLSQMWIGDK
β-Gal RCAWDQWRVPRALHPAPPPAQGVPPMLIEDDQGLTLTHGDQRWRFERSSGHLTQWWQNEQ

LacZ KQLLTPLRDQFTRAPLDNDIGVSEATRIDPNAWVERWKAAGHYQAEAALLQCTADTLADA
β-Gal PQLLTPLRDGFARAPIDNDIGVSEADHIDPNAWIERWKLAGLYRLEERCTQLQADALQNG

LacZ VLITTAHAWQHQGKTLFISRKTYRIDGSGQMAITVDVEVASDTPHPARIGLNCQLAQVAE
β-Gal VRVVSEHQFGVDGQILLISRKQWLFDALGAVSVNVEVEVADALPPPARIGLHCQLATVQP

LacZ RVNWLGLGPQENYPDRLTAACFDRWDLPLSDMYTPYVFPSENGLRCGTRELNYGPHQWRG
β-Gal QAEWLGLGPHENYPDRRLAAQYGRWRLPLAALHTPYIFPGENGLRCDTRSLRYGGWRIDG

LacZ DFQFNISRYSQQQLMETSHRHLLHAEEGTWLNIDGFHMGIGGDDSWSPSVSAEFQLSAGR
β-Gal RFHFSLSRYGLQQLMACSHQHLLQPEAGTWLHLDGFHMGVGGDDSWSPSVHRDYLLTAGV

LacZ YHYQLVWCQK---
β-Gal YRYQLRLQRAPEG

S2 Figure
