# Supplementary figures and images for: Functional metagenomics reveals novel β-galactosidases not predictable from gene sequences
Source: PLoS One. 2017 Mar 8;12(3):e0172545. doi: 10.1371/journal.pone.0172545 (PMC5342196; doi:10.1371/journal.pone.0172545)

A. glucose

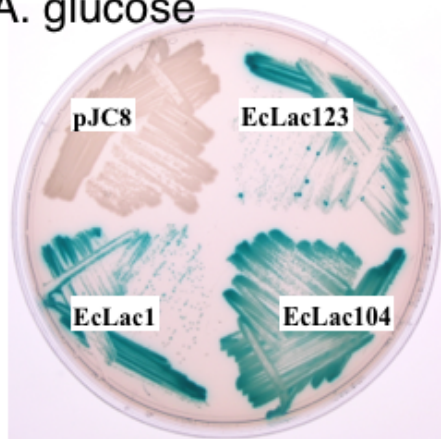

B. lactose

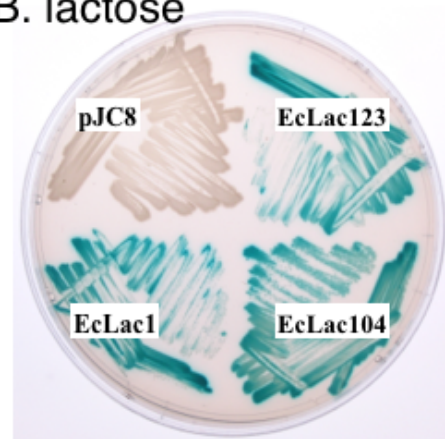

C. glycerol

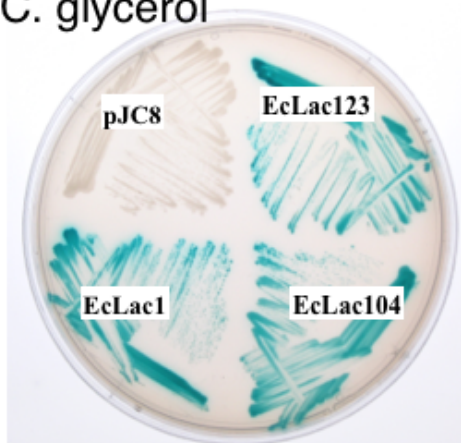

D. glycerol + IPTG

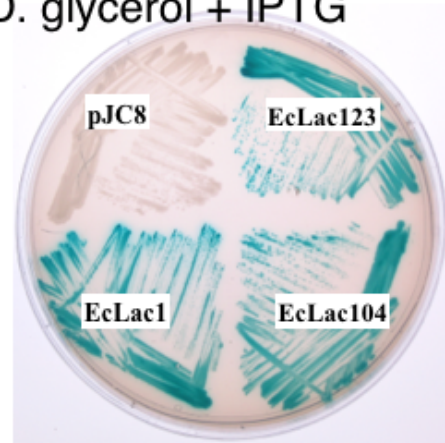

S3 Figure

Supplement: S3 File — Empty cosmid pJC8 was used as a negative control. X-Gal was used as chromogenic substrate. (A) M9-glucose (15 mM), (B) M9-lactose (15 mM), (C) M9-glycerol (30 mM) + 0.4 mM IPTG. (PDF) [file pone.0172545.s003.pdf]

A

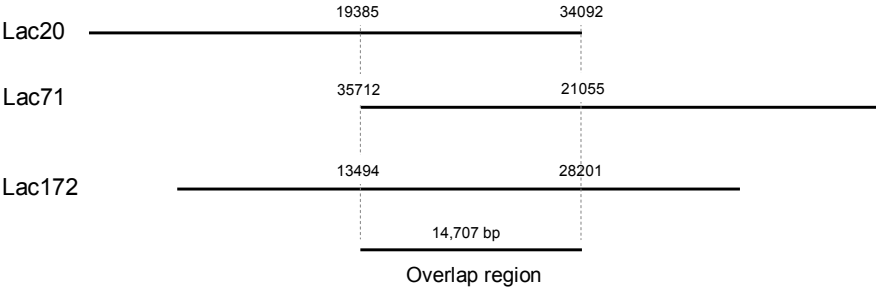

B

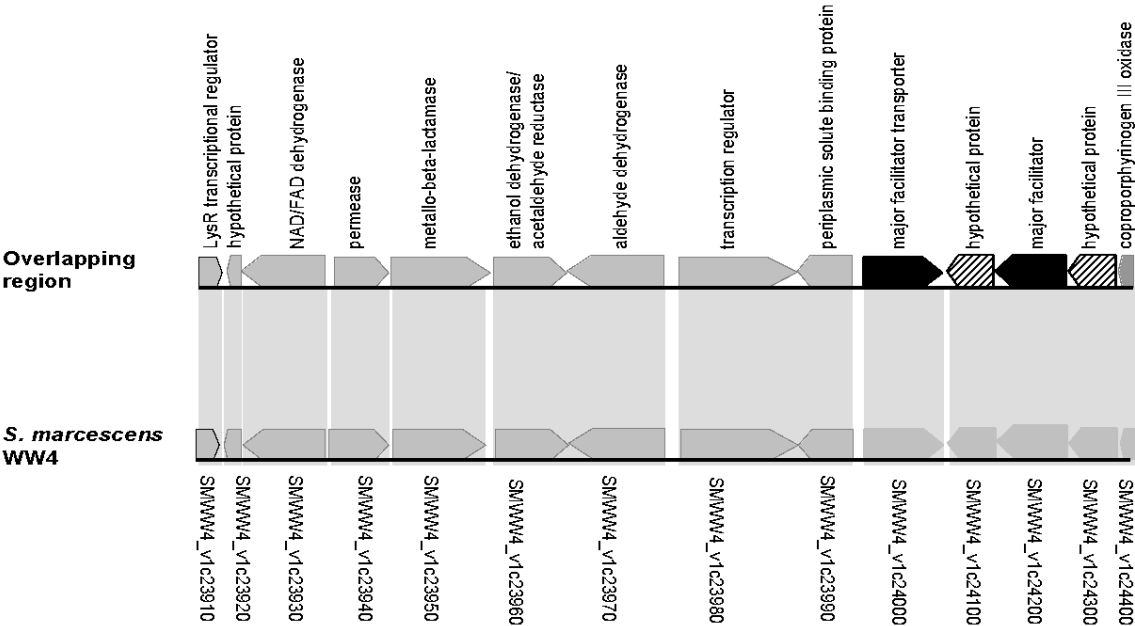

S4 Figure

Supplement: S4 File — (A) An overlapping region of 14,707 bp was present in those cosmids. (B) The major facilitator transporter(s) (solid box) in the region might be involved in lactose uptake. The hypothetical protein(s) (dash lined box) might be a β-galactosidase. Orthologs in γ-Proteobacteria Serratia marcescens WW4 chromosome (GenBank CP003959; 2,578,724–2,593,247 nt) were highlighted. (PDF) [file pone.0172545.s004.pdf]

A.

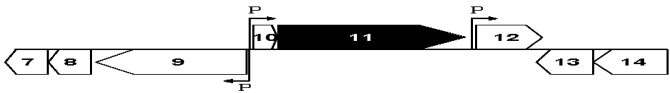

B.

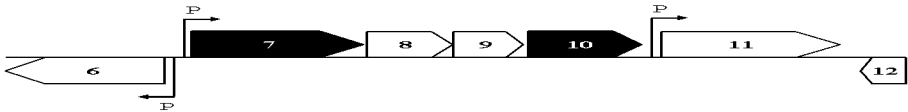

S5 Figure

Supplement: S5 File — (A) A gene locus from cosmid Lac36W (GenBank, KF255993). Lac36W_07, cytosine/adenosine deaminase; Lac36W_08, hypothetical protein; Lac36W_09, glutaminyl-tRNA synthetase; Lac36W_10, hypothetical protein; Lac36W_11, β-galactosidase; Lac36W_12, methionine-S-sulfoxide reductase; Lac36W_13, hypothetical protein; Lac36W_14, LysR family transcriptional regulator. The locations of potential promoter regions (P) were showed. (B) A gene locus from cosmid Lac161 (GenBank, KF255994). Lac161_06, histidine kinase; Lac161_07, β-galactosidase; Lac161_08, hypothetical protein; Lac161_09, hypothetical protein; Lac161_10, β-galactosidase; Lac161_11, hypothetical protein; Lac161_12, host specificity protein. The positions of potential promoter regions (P) are shown. (PDF) [file pone.0172545.s005.pdf]
