# Supplementary material for: Functional metagenomics reveals novel β-galactosidases not predictable from gene sequences
Source: PLoS One. 2017 Mar 8;12(3):e0172545. doi: 10.1371/journal.pone.0172545 (PMC5342196; doi:10.1371/journal.pone.0172545)
Supplement: S1 Table — (PDF) [file pone.0172545.s006.pdf]

S1 Table. DNA oligonucleotides used in this study with restriction recognition sites underlined.

Oligo ID	Sequence (5' to 3')
JC212	GAGTCATATGCCGACGCGCTGGCTCATC
JC213	AACACTCGAGGCTGAATTTCGCCGGGGCCACGCAGA
JC220	CGGGCATATGAGATTGTCGCCCAATCGCA
JC221	CGCGGTCGACGTGCCCCTCAGCAAAAATAA
JC226	CGCGCATATGCACCATCATCATCATC
JC227	GCGCGCTAGCATATAGTTCCTCCTTTCAGC
lac161NdeI	CGCCATATGGTGATTCCCATTGCGCGCAGACGG
lac161HindIII	CGGAAGCTTCATCGGACACGATCGGACGAACCG
