# Supplementary material for: Functional metagenomics reveals novel β-galactosidases not predictable from gene sequences
Source: PLoS One. 2017 Mar 8;12(3):e0172545. doi: 10.1371/journal.pone.0172545 (PMC5342196; doi:10.1371/journal.pone.0172545)
Supplement: S2 Table — (PDF) [file pone.0172545.s007.pdf]

S2 Table. β-Galactosidase activities of random 12AC *lac*$^+$ clones in *S. meliloti* RmF728. Cosmid pJC8 was used as a negative control. Clone numbers in bold font also complemented *E. coli* DH5α (Rif^R) in M9-lactose medium. Underlined clone numbers were completely sequenced. Whole cells of triplicate samples were permeabilized by SDS and reacted with ONPG. Specific activity was represented as (1000xA_{420})/(timexOD_{600} x culture volume).

**12AC *lac*$^+$ clone**	β-**Galactosidase specific activity ± standard deviation**
**12**	9902 ± 1052
16	4379 ± 643
**34**	15596 ± 1633
55	4388 ± 260
77	3958 ± 700
78	4301 ± 814
86	3226 ± 470
94	3292 ± 610
99	3671 ± 626
114	3558 ± 123
115	3240 ± 212
128	3596 ± 508
160	3094 ± 15
161	3916 ± 174
169	3606 ± 433
172	3903 ± 327
**177**	15445 ± 796
183	3331 ± 440
199	3851 ± 155
204	3357 ± 140
234	3589 ± 352
253	1801 ± 177
266	4001 ± 343
270	4351 ± 717
271	3835 ± 344
305	3679 ± 52
306	3770 ± 107
319	3788 ± 408
150B	3544 ± 633
150W	3889 ± 545
175B	4163 ± 582
195W	4211 ± 1099
203W	4112 ± 251
**206B**	15598 ± 1104
206W	4147 ± 309
**24B**	16973 ± 1348
267A	3707 ± 468
**35B**	16647 ± 664
36W	3772 ± 310
