# Supplementary material for: Functional metagenomics reveals novel β-galactosidases not predictable from gene sequences
Source: PLoS One. 2017 Mar 8;12(3):e0172545. doi: 10.1371/journal.pone.0172545 (PMC5342196; doi:10.1371/journal.pone.0172545)
Supplement: S3 Table — (PDF) [file pone.0172545.s008.pdf]

S3 Table. Top twenty homologs of Lac161_ORF10 detected by a BlastP search of the NCBI nr database.

Species	Protein (GenBank accession #)	Function	% identity	E-value	Bit score
Bacteroides sp. D22	gi:496330437	Conserved hypothetical protein	44.9	1.00E-145	444
Capnocytophaga sp. oral taxon 329 str. F0087	gi:725815884	Hypothetical protein	45.8	1.00E-145	444
Bacteroides finegoldii	gi:515710157	Hypothetical protein	44.5	2.00E-145	443
Bacteroides finegoldii CL09T03C10	gi:408473640	Hypothetical protein	44.5	4.00E-145	442
Chthoniobacter flavus	gi:494038897	Glycosyl hydrolase family 32 domain protein	47.3	8.00E-145	442
Bacteroides (multiple species)	gi:490423864	Hypothetical protein	44.1	1.00E-143	439
Bacteroides sp. 2_2_4	gi:229449608	Hypothetical protein	44.4	2.00E-143	438
Capnocytophaga sp. oral taxon 324	gi:496921710	Hypothetical protein	45.1	4.00E-143	437
Capnocytophaga sp. oral taxon 329 str. F0087	gi:725815883	Hypothetical protein	44.9	6.00E-143	437
Capnocytophaga sp. oral taxon 324	gi:496921709	Hypothetical protein	44.4	2.00E-141	434
Bacteroides eggerthii	gi:490420689	Hypothetical protein	43.1	5.00E-139	427
Capnocytophaga sp. oral taxon 326	gi:496931704	Hypothetical protein	43.9	2.00E-138	425
Bacteroides eggerthii CAG:109	gi:547198227	Conserved hypothetical protein	43.0	2.00E-138	425
Bacteroides (multiple species)	gi:496046067	Tat pathway signal sequence domain protein	44.4	3.00E-138	426
Bacteroides eggerthii	gi:490420690	Uncharacterized protein	44.7	1.00E-136	421
Bacteroides eggerthii CAG:109	gi:547198228	Uncharacterized protein	44.7	2.00E-136	421
Bacteroides sp. 3_1_23	gi:495921857	Conserved hypothetical protein	41.9	8.00E-135	417
Bacteroides eggerthii	gi:490418291	Hypothetical protein	43.0	5.00E-133	411
Bacteroides sp. 2_1_22	gi:262356909	Hypothetical protein	44.0	5.00E-132	410
Bacteroides xylanisolvens SD CC 2a	gi:292638199	Conserved hypothetical protein	44.0	7.00E-132	409
