# Supplementary material for: Functional metagenomics reveals novel β-galactosidases not predictable from gene sequences
Source: PLoS One. 2017 Mar 8;12(3):e0172545. doi: 10.1371/journal.pone.0172545 (PMC5342196; doi:10.1371/journal.pone.0172545)
Supplement: S4 Table — (PDF) [file pone.0172545.s009.pdf]

S4 Table. Detected abundance of three novel beta-galactosidases in a variety of metagenomic datasets.

#NOTE: Abundance of the taxonomic marker and housekeeping gene (RPOB) was also predicted for comparison.

BIOME	DATASET	**Lac161_10**	**Lac161_ORF7**	**Lac36W_ORF11**	**RPOB**
Aquatic	CAM_P_0000692_MILOCO.orfs.fa	92	3443	3033	191475
Aquatic	Antarctica_Aquatic_Microbial_Metagenome.orfs.fa	208	1629	10066	151451
Aquatic	CAM_PROJ_GOS.orfs.fa	65	921	3262	61673
Aquatic	CAM_PROJ_YLake.orfs.fa	69	987	2678	47527
Aquatic	CAM_P_0001026_LagrangianSamplingMontereyBay.orfs.fa	7	127	34	33957
Aquatic	ALOHA_orfs.fa	54	175	170	12165
Aquatic	CAM_PROJ_WesternChannelOMM.orfs.fa	10	129	452	10353
Aquatic	CAM_PROJ_BATS.orfs.fa	91	268	99	10349
Aquatic	CAM_P_0000719_MontereyBayTransect.orfs.fa	15	76	35	9893
Aquatic	CAM_PROJ_HOT_pyrosequenced.orfs.fa	17	93	104	8525
Aquatic	CAM_P_0001136_MahoneyLake.orfs.fa	209	179	2328	6145
Aquatic	CAM_P_0000545_Guaymas_Basin.orfs.fa	14	307	360	5098
Aquatic	CAM_P_0001028_NorthPacificMetagenomes.orfs.fa	14	1	24	4920
Aquatic	CAM_P_0001129_DeepChlorophyllMaximum.orfs.fa	2	35	13	4283
Aquatic	CAM_P_0000712_Bermuda_Oceanic.orfs.fa	69	175	70	4254
Aquatic	CAM_P_0001130_SantaPolaSaltern.orfs.fa	0	0	142	3995
Aquatic	CAM_PROJ_HOT.orfs.fa	39	95	77	3701
Aquatic	CAM_PROJ_Bacterioplankton.transeq.ORFs.fa	2	44	17	3586
Aquatic	CAM_PROJ_MontereyBay.orfs.fa	1	27	41	3286
Aquatic	CAM_PROJ_PacificOcean.orfs.fa	0	16	16	2628
Aquatic	CAM_PROJ_PML.orfs.fa	0	3	69	2480
Aquatic	CAM_PROJ_Sapelo2008.orfs.fa	0	28	28	2206
Aquatic	CAM_PROJ_AmazonRiverPlume.orfs.fa	0	6	22	2176
Aquatic	CAM_PROJ_GeneExpression.orfs.fa	0	6	17	1973
Aquatic	CAM_PROJ_BisonMetagenome.orfs.fa	3	9	496	1633
Aquatic	CAM_PROJ_IceMetagenome.orfs.fa	0	29	367	1503

Aquatic	CAM_P_0001133_HypersalineCoastalLagoon.orfs.fa	0	9	72	1415
Aquatic	Polar_Metagenome.orfs.fa	8	121	71	1309
Aquatic	CAM_P_0001132_FreshwaterLagoon.orfs.fa	10	67	219	1097
Aquatic	CAM_P_0001131_SantaPolaSaltern.orfs.fa	0	0	1	1094
Aquatic	Arctic_seawater_EBI_ERS089005.orfs.fa	9	88	46	915
Aquatic	CAM_PROJ_HypersalineMat.transeq.ORFs.fa	14	4	101	225
HumanGut	MH0012.fa	5	0	181	437
HumanGut	MH0006.fa	8	0	280	310
HumanGut	MH0009.fa	5	0	120	255
HumanGut	MH0086.fa	3	0	254	247
HumanGut	MH0050.fa	2	0	91	242
HumanGut	MH0082.fa	8	0	151	239
HumanGut	MH0011.fa	2	0	152	222
HumanGut	V1.CD-14.fa	1	0	137	219
HumanGut	MH0040.fa	7	0	110	217
HumanGut	V1.CD-8.fa	8	0	100	217
HumanGut	MH0081.fa	8	0	125	212
HumanGut	MH0069.fa	3	0	144	211
HumanGut	MH0065.fa	3	0	162	201
HumanGut	MH0042.fa	3	0	94	196
HumanGut	MH0070.fa	5	0	182	196
HumanGut	MH0054.fa	7	0	91	194
HumanGut	MH0060.fa	2	0	78	192
HumanGut	V1.CD-4.fa	2	0	140	190
HumanGut	MH0053.fa	0	0	103	189
HumanGut	MH0055.fa	3	0	161	188
HumanGut	MH0003.fa	5	0	125	187
HumanGut	MH0043.fa	4	0	92	187
HumanGut	MH0079.fa	3	0	29	184
HumanGut	V1.UC-19.fa	1	0	124	183

HumanGut	MH0077.fa	2	0	192	181
HumanGut	MH0080.fa	5	0	167	179
HumanGut	MH0059.fa	3	0	163	178
HumanGut	MH0075.fa	8	0	95	178
HumanGut	MH0031.fa	2	0	109	177
HumanGut	MH0066.fa	7	0	49	175
HumanGut	MH0025.fa	2	0	109	173
HumanGut	V1.UC-6.fa	5	0	183	173
HumanGut	MH0063.fa	2	0	102	171
HumanGut	V1.UC-9.fa	8	0	83	171
HumanGut	MH0035.fa	3	0	150	170
HumanGut	MH0083.fa	6	0	132	170
HumanGut	MH0033.fa	2	0	145	169
HumanGut	MH0038.fa	2	0	76	167
HumanGut	MH0014.fa	8	0	179	165
HumanGut	MH0058.fa	10	0	182	163
HumanGut	V1.UC-8.fa	5	0	124	161
HumanGut	MH0030.fa	2	0	82	160
HumanGut	MH0002.fa	1	0	90	156
HumanGut	MH0028.fa	5	0	127	155
HumanGut	MH0064.fa	15	0	124	155
HumanGut	MH0056.fa	10	0	89	154
HumanGut	MH0039.fa	5	0	146	152
HumanGut	V1.CD-13.fa	8	0	186	151
HumanGut	MH0071.fa	4	0	103	150
HumanGut	MH0016.fa	3	0	155	149
HumanGut	MH0041.fa	3	0	110	148
HumanGut	MH0036.fa	10	0	137	144
HumanGut	MH0044.fa	2	0	167	143
HumanGut	V1.CD-9.fa	1	0	52	142

HumanGut	MH0062.fa	5	0	101	140
HumanGut	V1.CD-11.fa	7	0	91	140
HumanGut	MH0020.fa	2	0	78	139
HumanGut	MH0057.fa	3	0	59	137
HumanGut	MH0067.fa	2	0	129	137
HumanGut	MH0052.fa	0	0	98	136
HumanGut	MH0076.fa	5	0	143	135
HumanGut	MH0045.fa	6	0	173	131
HumanGut	MH0068.fa	3	0	97	126
HumanGut	MH0074.fa	4	0	119	125
HumanGut	V1.CD-3.fa	1	0	86	125
HumanGut	MH0026.fa	1	0	98	123
HumanGut	MH0048.fa	3	0	62	123
HumanGut	MH0051.fa	1	0	78	120
HumanGut	MH0061.fa	7	0	82	120
HumanGut	MH0032.fa	1	1	104	118
HumanGut	MH0021.fa	5	0	108	118
HumanGut	MH0037.fa	5	0	69	118
HumanGut	MH0085.fa	8	0	76	115
HumanGut	MH0073.fa	5	0	142	111
HumanGut	MH0046.fa	4	0	98	109
HumanGut	MH0047.fa	1	0	16	105
Soil	4510219.3.transeq.fa	1001	732	7714	384993
Soil	4541646.3.transeq.fa	498	833	1596	74631
Soil	4541645.3.transeq.fa	544	990	1831	71208
Soil	4541647.3.transeq.fa	474	800	1783	61175
Soil	4541642.3.transeq.fa	465	771	1724	60466
Soil	4541649.3.transeq.fa	272	652	1738	55281
Soil	4539063.3.transeq.fa	343	924	1515	53483
Soil	4541651.3.transeq.fa	304	557	2099	51570

Soil	4541641.3.transeq.fa	356	665	1378	50646
Soil	4539064.3.transeq.fa	393	652	1545	48747
Soil	4541644.3.transeq.fa	347	623	1572	47658
Soil	4541648.3.transeq.fa	279	564	1138	47348
Soil	4541650.3.transeq.fa	305	722	1765	47232
Soil	4543020.3.transeq.fa	5	52	185	3671
Soil	4514245.3.transeq.fa	4	11	204	3317
Soil	4543023.3.transeq.fa	3	58	172	2716
Soil	4543022.3.transeq.fa	1	24	68	2490
Soil	4446153.3.transeq.fa	22	156	234	1238
Soil	4543019.3.transeq.fa	0	5	66	981
Soil	4543021.3.transeq.fa	1	14	36	980
Soil	4537193.3.transeq.fa	5	67	317	952
Soil	4537195.3.transeq.fa	10	108	262	878
Soil	4537194.3.transeq.fa	3	60	177	777
Soil	4537190.3.transeq.fa	7	95	249	773
Soil	4537191.3.transeq.fa	9	64	146	678
Soil	4537192.3.transeq.fa	1	38	177	651
Soil	4478941.3.transeq.fa	1	23	95	322
Soil	4478940.3.transeq.fa	5	31	131	278
Soil	4478294.3.transeq.fa	0	17	87	275
Soil	4478038.3.transeq.fa	1	20	81	272
Soil	4479311.3.rhiz.transeq.fa	3	22	120	271
Soil	4478937.3.transeq.fa	2	32	103	255
Soil	4477790.3.transeq.fa	2	29	81	253
Soil	4478290.3.rhiz.transeq.fa	1	29	114	253
Soil	4478939.3.rhiz.transeq.fa	2	29	105	249
Soil	4477749.3.rhiz.transeq.fa	0	26	103	243
Soil	4477751.3.rhiz.transeq.fa	3	23	89	242
Soil	4478934.3.rhiz.transeq.fa	0	10	93	239

Soil	4478291.3.rhiz.transeq.fa	3	17	86	237
Soil	4478222.3.transeq.fa	0	24	71	234
Soil	4478283.3.transeq.fa	0	21	117	232
Soil	4478030.3.rhiz.transeq.fa	0	20	84	231
Soil	4478936.3.transeq.fa	4	26	99	216
Soil	4477755.3.rhiz.transeq.fa	4	28	99	211
Soil	4478943.3.transeq.fa	3	25	97	209
Soil	4477757.3.transeq.fa	3	28	73	207
Soil	4478037.3.rhiz.transeq.fa	0	17	73	206
Soil	4478938.3.rhiz.transeq.fa	8	15	113	204
Soil	4478292.3.rhiz.transeq.fa	5	25	103	188
Soil	4477789.3.transeq.fa	0	24	53	176
